# Managing a Mandibular Second Premolar with Three-Canal and Taurodontism: A Case Report

**Published:** 2013-01-20

**Authors:** Hadi Mokhtari, Mahdi Niknami, Vahid Zand

**Affiliations:** 1 Department of Endodontics, Dental School, Tabriz University of Medical Sciences, Tabriz, Iran; 2 Department of Oral and Maxillofacial Radiology, Dental School, Tehran University of Medical Sciences, Tehran Iran

**Keywords:** Cone-Beam Computed Tomography, Taurodontism, Tooth Abnormalities

## Abstract

Root canal anatomy diversity and aberrations are common especially in permanent dentition. A thorough understanding of the basic root canal anatomy and its diversities are necessary for successful endodontic treatment. Mandibular second premolars are usually single-rooted and have one root canal. Incidence of three separate root canals in this tooth is quite rare and taurodontism with three separate canals has never been reported in literature so far. The use of cone-beam computed tomography scan in this rare case greatly contributed to making a confirmatory diagnosis and successful nonsurgical endodontic management thereafter.

## 1. Introduction

A proper understanding of the anatomy of root canal system is absolutely necessary for successful endodontic treatment [1, 2]. The main objective of endodontic treatment is accurate mechanical and chemical debridement of the entire root canal system, which should be followed by a three-dimensional obturation with an appropriate filling material and a final coronal restoration to prevent microleakage [2]. Thorough knowledge about the occurrence of unusual external and internal root canal morphologies contributes to successful root canal treatment.

Dental anomalies constitute great part of tooth morphology variations; one of the most important abnormalities in tooth morphology is taurodontism.

Taurodontism can be defined as a change in tooth shape caused by the failure of Hertwig’s epithelial sheath diaphragm to invaginate at the proper horizontal level. An enlarged pulp chamber, apical displacement of the pulpal floor, and no constriction at the level of the cementoenamel junction are the characteristic features. The wide range of variability of prevalence [from less than 0.1% to 48%] is most likely because of different diagnostic criteria and racial variations [3].

Literature reviews of this subject reveal wide variations and diversities in the root canal morphology of mandibular premolars.

Among different studies carried out on aberrations of root canal system [4-9], Zillich and Dowson and also De Moor [8, 9] reported mandibular second premolars with three canals. Vertucci reported that the second premolars have only one root canal at the apex in 97.5% of the teeth under study and two canals were only found in 2.5% of cases; the incidence of three root canals was extremely rare [8, 10].

Rodig and Hulsmann [2]reported a case of mandibular second premolar with three separate roots and root canals which were diagnosed using intraoral periapical radiographs. Wong [11], Bram and Fleisher [12], and Al-Fouzan [13] studied mandibular second premolars with four canals. Tzanetakis et al. performed endodontic treatment on a mandibular second premolar with four root canals (the canals wer discovered with the aid of an operating microscope) [14].

Suchdeva et al. have concluded that the occurrence of four separate roots with four distinct root canals in the mandibular second premolars has never been reported in the literature [15]. Vujaskovic et al. have reported a case of taurodont second premolar [16] and some others documented cases of taurodont molars [17, 18].

This rare case report presents successful nonsurgical endodontic treatment of a taurodont mandibular right second premolar with three separate root canals, wherein cone-beam computed tomography (CBCT) was used as a confirmatory diagnostic tool.

## 2. Case Report

A 23-year-old male patient referred to our office with a chief complaint of intermittent pain in relation to their premolar tooth (mandibular right second premolar) for approximatgely 3-months. The patient also complained of episodes of sensitivity to hot and cold food in the involved tooth. Medical history was noncontributory.

**Figure 1.**
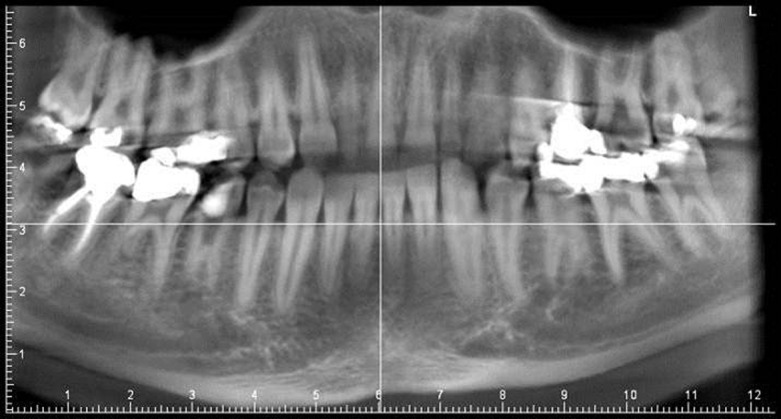
Panoramic view shows the vague outlines of taurodont premolars in the both side. Peg-shaped maxillary right lateral incisor and bilateral three-root mandibular first molars were also observed

Clinical examination revealed moderate oral hygiene; also there was a temporary filling in tooth #29, with secondary caries around the margins. There was no evidence of either swelling or sinus tracts. The involved tooth was tender to percussion. No periodontal pockets were present. Radiographic evaluation of the involved tooth revealed an unusual, complex root canal anatomy. Vague outlines of two roots with taurodontism could be identified. There was widening of the periodontal ligament space with periapical radiolucency around the distal root of tooth #29. In the panoramic radiography, the same condition was observed on tooth #20. In addition, the peg-shaped tooth #7 (maxillary right lateral incisor) and bilateral three-root mandibular first molars were seen (Figure 1).

Based on clinical and periapical radiographic findings, the patient was referred to an oral and maxillofacial radiologist for a cone-beam computed tomography. Informed consent was obtained from the patient and a CBCT of the mandible was performed by using the Promax3D (Planmeca, Helsinki, Finland). A three-dimensional image of the mandible was obtained. The involved tooth was focused, and the morphology was obtained in transverse, axial, and sagittal sections with a thickness of 0.48 mm, along with three-dimensional reconstructed images.

**Figure 2.**
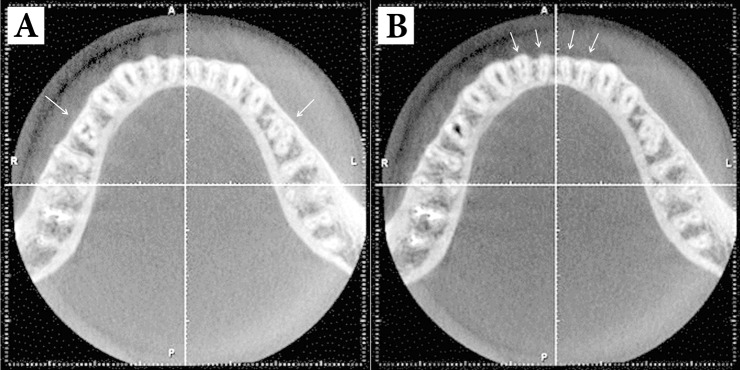
Axial images of CBCT presenting: A: Bilateral three-root mandibular second B: Second canals of all mandibular incisors are visible

The axial images of CBCT revealed other variations such as third canals in taurodont premolars and second canals in all mandibular anterior teeth (Figure 2A, 2B).

Subsequent to the confirmatory diagnosis, a treatment plan was prepared for the involved tooth. After local anesthesia was administered, a rubber dam was used to isolate the tooth. The defective restoration and secondary caries were removed. The access opening was modified with great attention to the approximate locations of the orifices of the three root canals.

**Figure 3.**
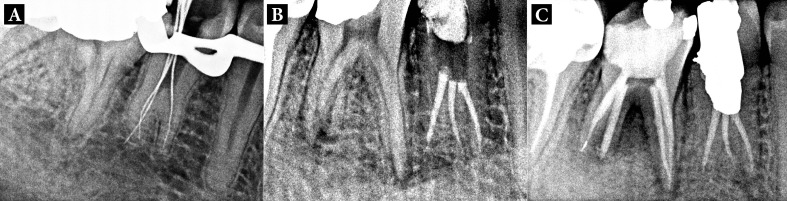
Periapical radiographs of treatment procedures. A: Working length determination. B: Final obturation. C: Healing after 1-year follow-up. Note that during this time, RCT of mandibular right first molar was performed

On careful inspection of the pulp chamber floor, three separateroot canal orifices were detected (one mesiobuccal, one mesiolingual, and one distal). Coronal flaring of all the three canals was carried out with Gates Glidden drills and working length was determined using an apex locator (Root ZX; Morita, Tokyo, Japan), which was later confirmed by a radiograph (Figure 3A).

The canals were cleaned and shaped up to ISO #35 master apical file under copious irrigation with 2.5% sodium hypochlorite and 17% EDTA. The root canals were dried with sterile paper points (Dentsply, Maillefer, Ballaiques, Switzerland) and filled with calcium hydroxide paste (Calcicur; VOCO, Cuxhaven, Germany); then, access cavity was temporarily sealed with Cavit (3M ESPE AG, Seefeld, Germany). The patient was scheduled for one-week follow up. The tooth was completely asymptomatic at this follow-up. Calcium hydroxide paste was removed, and the roots canals were obturated by cold lateral compaction of gutta-percha using AH26 sealer (Kemdent; Associated Dental Products Ltd, Wiltshire, UK). A postoperative radiograph was taken (Figure 3B), and the access cavity was restored permanently with universal amalgam restorative material. A 1-year recall radiograph showed satisfactory healing. During follow-up period, root canal treatment was also performed on tooth #30 (Figure 3C).

## 3. Discussion

Diagnosis and management of extra roots and root canals in mandibular premolars is one of challenges in endodontics [19-29]. Therefore, the clinician must have a proper knowledge of the normal root canal anatomy and of its most common variations. Inability to find, debride and obturate a root canal has been reported to be a major reason for failures in endodontic treatment [30]. Hoen and Pink reported an incidence rate of 42% for missed roots or canals in the teeth that needed retreatment [31]. Hence, it is of utmost importance that all the canals be located and treated during the course of nonsurgical endodontic treatment. Mandibular premolars have a reputation of having an aberrant anatomy [32]. Different studies have reported a relatively high prevalence rate for mandibular premolars with more than one root canal [33, 34-36]. Considering the high prevalence of aberrations in these teeth, the endodontist must suspect the presence of missed canals when a patient returns with post-operative persistent pain or sensitivity to hot and cold. Judicious use of high-end diagnostic aids should also be considered in such cases.

Radiographs yield two-dimensional images of three-dimensional objects, resulting in superimposition of images. Therefore, they are of rather limited use and value in complex root canal anatomy Cases.

Interpretation based on a two-dimensional radiograph may alert the clinician to the presence of aberrant anatomy but cannot fully show the morphological structure of root canals and their interrelations [37]. Based on the results of previous studies carried out by Kottoor et al., and La et al. wherein spiral CT was used for the confirmatory diagnosis of morphological aberrations in the root canal anatomy, CBCT of the involved tooth was planned in the present case [38-40].

The CBCT images in this study revealed two separate taurodont roots (one mesial and one distal) with three distinct root canals. Each of the root canals had separate apical orifice. Although the vague outlines of the two roots could be observed on the radiograph, the confirmatory diagnosis of the three root canals (mesiolingual), third canal of mandibular left second premolar and also second canals of all mandibular anterior teeth could only be made with the help of CBCT.

Clearly, these findings are clinically important as in a study at the University of Washington assessing the results of endodontic therapy, in which the mandibular first and second premolars showed failure rates of 11.45% and 4.54% [41]. Conceivably, these findings might be attributed to the complex root canal anatomy of a large number of these teeth. Numerous opinions have been reported in literature regarding the number of root canals, but there are very few reports on the variations in the number of roots in mandibular premolars [42, 43].

During root canal treatment of taurodont teeth, the clinician should appreciate the complexity of the root canal system, canal obliteration and configuration, and the potential for extra roots and canals. Careful exploration of the grooves between all the orifices, particularly with magnification, use of ultrasonic irrigation, and a modified filling technique are particularly important [3].

Successful nonsurgical endodontic treatment of a taurodont mandibular second premolar with three separate root canals was presented. In this case, the presence of a third root canal in a taurodont tooth and also other variations in the root canal system were diagnosed by CBCT.

## 4. Conclusion

In this case, exact root canal anatomy could not be depicted by using periapical radiographs alone. Therefore, the use of CBCT helped us make a confirmatory diagnosis. The success of this case might be attributed to accurate diagnosis, complete chemo mechanical debridement, and proper obturation of all the three root canals.
